# Human cadaver decomposition islands and forensic taphonomy: gravesoil δ^13^C and δ^15^N enrichment patterns in short (30 d) and extended (900 d) postmortem intervals

**DOI:** 10.1093/fsr/owaf027

**Published:** 2025-10-15

**Authors:** Kelly L Miles, Victoria Gibbon, Brian Hayden

**Affiliations:** Department of Biology, University of New Brunswick, New Brunswick, Canada; Department of Human Biology, University of Cape Town, Cape Town, South Africa; Department of Biology, University of New Brunswick, New Brunswick, Canada

**Keywords:** forensic sciences, forensic taphonomy, human decomposition, gravesoil, cadaver decomposition island, postmortem interval (PMI), stable isotopes

## Abstract

Soil forensics examines edaphic properties of evidentiary value in the immediate vicinity of human remains in a region called the cadaver decomposition island (CDI). As a body decomposes, soil in the CDI is permeated with liquified human nutrients and becomes gravesoil. This study is the first to specifically investigate how stable isotopes of carbon (δ^13^C) and nitrogen (δ^15^N) change in human gravesoils during decomposition over the postmortem interval (PMI). Gravesoil samples were analysed for %C, δ^13^C, %N, and δ^15^N in CDIs over the initial 30-d PMI (PMI_30_) and over longer PMIs up to 900 d (PMI_900_). Gravesoil %C and δ^13^C did not show any significant changes with decomposition progression in the PMI_30_ study. By days 10–15, gravesoil mean %N more than doubled from 0.4% to 1.05%, while mean gravesoil δ^15^N increased from 3.3‰ to 8.2‰ by day 10, up to a high mean of 9.9‰ on day 20. Both %N and δ^15^N remained significantly elevated for the entire PMI_30_ period. In the PMI_900_ study, gravesoils increased from 0.2%–0.8% N to a high of 1.9% N after 2–3 months, then decreased to 0.5%–0.6% in the 600–900 d time frame. Gravesoil δ^15^N originating at 2‰–4‰ was enriched by 15‰–20‰ after the first month, eventually declining to 9‰–11‰ after 600–900 d. We conclude that δ^15^N in human gravesoils has promise as a forensic tool to detect a CDI and correlate it with decomposition stage that adds to existing methods in estimating PMI, even if the body is absent.

**Key Points**
 

- Soil forensics examines the products of human decomposition and their impacts on underlying gravesoil.
- Various forms of nitrogen and carbon have proven to be a useful indicator of the presence of gravesoils.
- %C, δ^13^C, %N, and δ^15^N in CDIs were measured at different depths and distances from the remains in the short PMI (30 d) and extended PMI (900 d).
- When compared to control soils, δ^15^N and %N levels in gravesoil increased by day 10, peaked by day 20, and remained high over the 30-d study. In the longer PMI study, %N returned to control levels in the 600–900 d time frame, while δ^15^N levels remained elevated at the end of the experiment.
- We conclude that δ^15^N is a useful indicator of not only the presence of a CDI but also has tangible use as a PMI estimation tool for forensic taphonomists.

## Introduction

Forensic taphonomists study mechanisms of human decomposition, in part, to provide law enforcement with accurate and reliable postmortem interval (PMI) estimations using both qualitative and quantitative assessment of decomposing tissues [1–4]. Multi-disciplinary collaborations have expanded their focus from modelling the environment’s impact on cadaver decomposition [5] to instead examine how tissue decomposition impacts and changes the surrounding environment with particular attention paid to gravesoils under a body [6, 7]. Whereas gravesoils have previously had value as trace evidence in criminal investigations [8], the biochemical impacts of a decomposing body are being recognized as having meaningful and quantifiable impacts on gravesoils with potential forensic significance [9–15].

In recent decades, gravesoil science examined the potential for soils under a decomposing body to reveal information about PMI [9, 13, 16]. The area where decompositional body fluids have infused into soils beneath a body, termed a cadaver decomposition island (CDI), experiences a significant input of body-derived nutrients in the early PMI, particularly in carbon and nitrogen. Over time, the amounts of varying forms of carbon and nitrogen in the CDI gravesoil will eventually decrease back to levels seen before the decomposing body was present [11, 15].

Benninger et al. [17] monitored CDI gravesoils under decomposing porcines for 100 d and detected total nitrogen increased from ~0.25% to 0.6% between days 21 and 42. Gravesoil returned to ambient total nitrogen levels by day 100. Carcass fluids from decomposing porcines were found to significantly elevate gravesoil ninhydrin-reactive nitrogen (NRN) levels after 4 d of decomposition [14]. Van Belle et al. [15] reported gravesoils increased in NRN from 20 to 90 μg N/g soil by days 10–14 and remained elevated beyond the end of the 100-d study. With decomposing rats (*Rattus rattus*), NRN was similarly elevated in gravesoils at day 7 and remained elevated at the end of the 28-d experiment [10].

Complementing these previous short-term studies, Fancher et al. [11] published long-term data for human CDIs compared to control soils of ~6 μg N/g soil where ammonium nitrate (NH_4_-N) levels in CDIs were significantly higher 14-d after decomposition (111 μg N/g soil), while nitrate nitrogen (NO_3_-N) increased from 36 μg N/g soil to 245 μg N/g soil by 171-d. These measures of nitrogen returned to control levels after 732 and 851 d, respectively.

Determining the extent and rates that cadaver nutrients from liquefied decomposing tissues permeate nearby gravesoils, both vertically and horizontally in CDIs, has only been recently investigated. CDIs were examined at 20 cm and 50 cm radius intervals around decomposing porcines in Canada [13]. Carcass nutrients were elevated compared to control soils at a 20 cm radius for phosphorus and sodium by 195 accumulated degree days (ADD). Adult human bodies placed on surface soil plots of 1 m × 2.5 m in Texas showed significantly elevated levels of dissolved organic carbon and nitrogen after 248–288 d of decomposition along a distance gradient that extended out to the periphery of the plot boundaries [9]. Fancher et al. [11] sampled CDI gravesoils from 0 to 15 cm depths under donor bodies in two separate locations with different soil types. Surface gravesoils showed significantly more dissolved organic carbon (DOC) in gravesoils by day 6, peaking at days 14 and 132 for the two soil types, while dissolved organic nitrogen (DON) peaked at days 132 and 263 for the two soil types. Both DOC and DON remained elevated over the course of the study. When examining deeper layers of the same gravesoils, depths up to 15 cm under the body also showed increased DON, NH_4_-N and NRN, albeit delayed in timing and to a lesser degree compared to surface CDI gravesoils. There are still many unanswered questions about cadaver nutrient influx in CDIs in other avenues of forensic taphonomy of gravesoils not yet studied, namely soil stable isotope analysis.

The influence of stable isotopes of carbon and nitrogen from decomposing human tissues on CDI gravesoils is currently unknown, but mammal data provide insight into this question. Keenan et al. [12] report gravesoils under decomposing beaver (*Castor canadensis*) carcasses (“carrion hotspots”) had elevated levels of total nitrogen and δ^15^N (by (7.5 ± 1.0)‰) a year after deposition. A longitudinal study of temporal and spatial human gravesoil enrichments of stable isotopes has not yet been published but would provide forensic data conspicuously lacking in the literature. If such an enrichment pattern can be detected and monitored over the decomposition progress (and beyond skeletonisation), it would be a huge step forward in validating the use of stable isotopes in a new aspect of CDI forensic case work.

The aim of this study is to investigate the liberation of both δ^13^C and δ^15^N from liquefied human tissues through natural outdoor decomposition into CDI gravesoils in order to answer three main questions based on current gaps in forensic taphonomy: (1) Is there a quantifiable increase in δ^13^C and δ^15^N in CDI gravesoils over time, and if so, at what point in the early PMI is this enrichment first detectable, (2) do detectable levels of these increasingly enriched stable isotopes differ with greater lateral and horizontal distances from the body, and (3) how long do these enrichments persist over extended PMI periods? If significant, these temporal and spatial data could provide meaningful information that adds to our understanding of CDI gravesoil dynamics as well as supporting the use of stable isotopes as another tool that can be used for PMI estimates in forensic casework.

## Materials and methods

Research was conducted at the Forensic Anthropology Research Facility (FARF), in San Marcos, Texas. FARF occupies 26 acres of secured and fenced outdoor area within Freeman Ranch, an agricultural research facility and working farm managed by Texas State University, San Marcos. Soil at FARF has a dark brown, high clay-content topsoil that overlies limestone, is significantly rocky or cobbled, has low moisture permeability, and neutral pH [18].

Body donations are obtained through a voluntary donation programme with the express documented permission of the deceased individual and/or the consent of the next of kin for the purposes of forensic research, managed by the Forensic Anthropology Centre for Taphonomic Research (FACTS) at Texas State University (Research and Ethics Request approval number: 04–039-001). Donor cadavers are placed under exclusion cages to prevent large scavenging activity by avian or canid animals but still permit free access to sunlight, airflow, and insects as normal taphonomic processes [19]. The day the donor body was placed in the FARF outdoor facility was considered day 0 for the experiment, as some donors were brought to the facility from a distance and required overnight refrigeration at 4 °C before placement in the field the following day.

### Soil sampling

#### Gravesoils sampled during the early PMI period from days 0–30 (PMI_30_)

**Figure 1:**
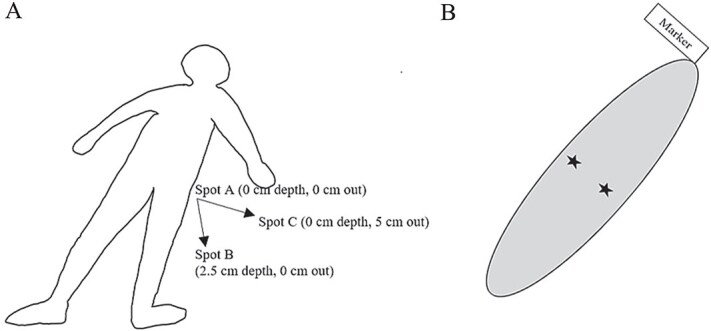
Diagrams of (A) prone donor body in the early PMI (PMI_30_) gravesoil study, and (B) wooden marker at the head of a cadaver decomposition island (CDI) for the extended PMI (PMI_900_) gravesoil study in San Marcos, Texas. Spots A, B, C represent sampling spots within the short period PMI_30_ CDIs near a donor, while black stars indicate extended period PMI_900_ sampling CDI locations without donor remains present. PMI: postmortem interval.

Six donor bodies were placed at FARF and permitted to decompose naturally under exclusion cages to obtain data for the early PMI from 0–30 d. At 5-d intervals, samples were taken from gravesoils in three locations in each CDI: Spot A on the surface soil directly under the donor’s hip (0 cm depth, 0 cm out); Spot B was 2.5 cm under the surface soil and directly by the donor’s hip (2.5 cm depth, 0 cm out), and Spot C on the surface soil at a distance 5 cm out from the body’s hip (0 cm depth, 5 cm out; Figure 1A). Samples for these locations were repeated on the left and right sides of the body as duplicates on each sampling day. The groin/hip region of the body was selected due to its ease of access for sampling underlying gravesoil as well as the likelihood it would contain a significant amount of liquefied nutrients from the body. This region is often the first and most obviously impacted during the decomposition process due to overlying abdominal putrefaction. Samples were not collected at additional depths past 2.5 cm due to significant underlying rock. Control samples were obtained from soils where bodies had never been placed and used for comparisons with the PMI_30_ soil samples.

For sample spots A and C, the surface substrate was gently cleared away to remove obvious vegetation and leaf litter to ensure only soil was collected. Spot B soil was disturbed as little as possible to obtain accurate samples at 2.5 cm depth using a ruler, and sampling points were not reused; each sampling day created a new alteration to the soil surface.

#### Gravesoils sampled over an extended PMI of up to 900 d (PMI_900_)

In most cases, the visual evidence of the CDI remains long after the body has been removed due to soil staining. This allowed for sampling of shorter PMI gravesoils where bodies were still present, as well as extended PMI gravesoils where the donor body had been removed upon skeletonisation. Sampling of the extended period PMI_900_ gravesoils involved clearing away the surface vegetation and leaf litter from an area ~10 cm to the left and right of the centre of the CDI to approximate where the groin of the donor would be located (Figure 1B). This was done with replicates for as many different donor CDIs with varying PMIs as possible. Control samples were obtained from soils where bodies had never been placed and used for comparisons with the PMI_900_ soil samples.

For both the early PMI and extended PMI studies, each sample removed ~1 cm^3^ of soil from its respective location by use of a small gardening spade and a lab-grade curved spatula-like scoop. For the PMI_900_ study, a ruler was then used to measure the 2.5 cm depth from the surface soil from the small separations made with the spade. Soil was secured in individual 1.5 mL Eppendorf tubes with snap-caps then immediately stored in a –20 °C freezer. The spade and spatula scoop were thoroughly rinsed with distilled water and paper lab wipes between samples to minimize cross-contamination of samples.

### Stable isotopes- δ^13^C and δ^15^N

Frozen sample tubes were allowed to thaw at room temperature with open caps for 3 h, and then housed in a drying oven at 60 °C for a minimum of 48 h to completely desiccate the samples rendering them biologically inert for isotopic analysis. Dried samples were pulverized to a uniform powdered consistency and lipid were extracted to avoid potential influence on carbon stable isotope ratios. Lipid extraction submerged the powdered samples in a 2:1 chloroform to methanol solution for a minimum of 30 min, repeated at least three times or until the supernatant was clear and colourless. Stable isotopes were analysed using continuous flow isotope-ratio mass spectrometry (CFIRMS) to obtain values of ^13^C and ^15^N. These absolute values were then reported as relative values of δ^13^C and δ^15^N when compared to universal standards of Vienna Pee-Dee Belemnite (V-PDB; [20]) and atmospheric air (AIR; [21]), respectively. Four in-house secondary standards, Corn Meal Standard (δ^13^C: −13.3‰, δ^15^N: 14‰), Ephedra Plant Standard (δ^13^C: −30.9‰, δ^15^N: 0.4‰), Spirulina Standard (δ^13^C: −24.9‰, δ^15^N: 12.9‰), and Seaweed Plant Standard (δ^13^C: −28.4, δ^15^N: 21.1‰), calibrated against International Atomic Energy Agency (IAEA) standards, were used to normalize stable isotope values. To assess analytical accuracy, check standards of acetanilide, N_2_ and CH_7_ were also analysed. Furthermore, 5%–7% of samples were run with replicates to monitor instrument drift over time. Analytical precision was below 0.3‰ for both δ^13^C and δ^15^N following repeated analysis.

Statistical analysis was performed using R [22] to determine significant changes in %C, δ^13^C, %N, and δ^15^N for several comparisons: the general linear model for an effect of decomposition progression (days), and paired Student’s *t*-tests to compare between sampling days for each isotope at each sampling location during the early PMI study. Significance was established at α = 0.05.

## Results

Elemental and isotopic composition of soil collected at CDIs revealed a significant influence of liquefied human tissue nutrients into adjacent gravesoils. While carbon showed highly variable results for both PMI_30_ and PMI_900_ gravesoils, rapidly detectable elevated measures of mean %N and δ^15^N in the initial PMI_30_ phase persisted well into the extended PMI_900_ study.

### Gravesoils sampled during the early PMI period from days 0–30 (PMI_30_)

CDI soils exposed to decompositional fluids in the first 30 d showed different results for both elemental and isotopic measurements of carbon and nitrogen. General linear model (GLM) analysis did not identify a statistically significant relationship between decomposition progression and either mean soil %C or mean soil δ^13^C (Table 1). Mean measures for nitrogen, however, showed distinct increases over time. Decomposition affected the mean %N at Spots A (0 cm down, 0 cm out) and B (2.5 cm down, 0 cm out), but no such effect was detected for Spot C (0 cm down, 5 cm out). Mean δ^15^N, however, was significantly impacted by the decomposition process over time for all three soil spots sampled.

**Table 1:** Summary of general linear model *P* values for five mean gravesoil measurements for three sampling spots in six human cadavers’ grave soils over the first 30 d of outdoor decomposition (PMI_30_) at the forensic anthropology research facility, San Marcos, Texas.

Mean soil measure	Spot A (0 cm down; 0 cm out)	Spot B (2.5 cm down; 0 cm out)	Spot C (0 cm down; 5 cm out)
δ^15^N	<0.0001	<0.0001	0.03
%N	0.006	0.003	-
δ^13^C	-	-	-
%C	-	-	-
C:N	0.01	-	-

-: no significant effect of time (and therefore decomposition progression) was detected (*P* > 0.05).

**Figure 2:**
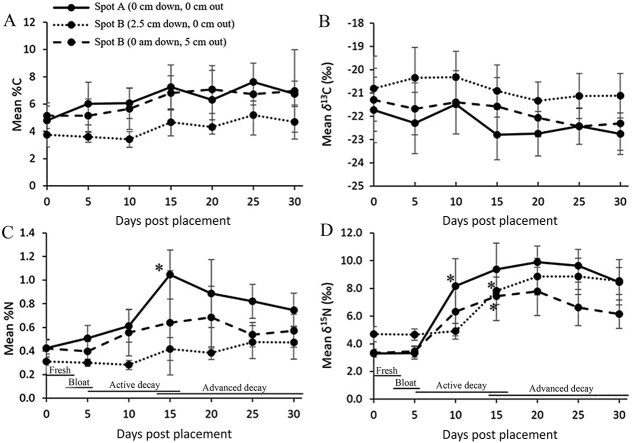
Mean (± standard error) values of (A) percent carbon (%C), (B) δ^13^C (‰), (C) percent nitrogen (%N), and (D) δ^15^N (‰) for three sampling spots adjacent to six human cadavers’ grave soils over the first 30 d of outdoor decomposition (PMI_30_) at the Forensic Anthropology Research Facility, San Marcos, Texas. Asterisk denotes the first point at which a significant mean increase was detected compared to day 0, *P* < 0.05.

Each sampling day’s gravesoil mean measurement was compared to that of the starting day soils at day 0. No discernible enrichment pattern of gravesoils was found with either %C or δ^13^C over time except for a single point of enrichment for %C at Spot A on day 25 (Figure 2A and B).

Unlike carbon gravesoil data, clear patterns emerged with %N and δ^15^N. The first point in time when mean %N was significantly higher compared to day 0 gravesoil was detected on day 15 for the Spot A samples, corresponding to the end of active decay and beginning of advanced decay phase of cadaver decomposition. Mean %N increased from 0.43% on day 0 to 1.05% by day 15, more than doubling the initial total nitrogen gravesoil content. Spot B samples were significantly higher in %N as well but detectable much later, first at day 30 well into the advanced stage of cadaver decomposition. This increase in nitrogen at Spot B was less pronounced than that at Spot A, with a day 30 mean percent nitrogen of 0.49% N compared to 0.31% at day 0. Samples collected from Spot C were not found to be higher in %N at any point in the 30-d study period (Figure 2C).

Mean values for Spot A δ^15^N gravesoils showed significant enrichments at day 10 in the active decay stage of decomposition, 5 d earlier than the first elevated levels of mean %N were detected. The original day-0 mean δ^15^N of 3.3‰ increased to 8.2‰ at day 10, an overall increase of 4.9‰ that remained significantly elevated throughout the 30-d study. In addition to Spot A, gravesoils at Spots B and C also had significantly elevated mean δ^15^N levels detectable at day 15. Spot B’s mean δ^15^N at day 0 of 4.7‰ increased to 8.0‰ by day 15 (an increase of 3.3‰), while Spot C’s day 0 mean δ^15^N of 3.3‰ increased to 7.4‰ in the same 15-d period (an increase of 4.1‰) (Figure 2D).

**Figure 3:**
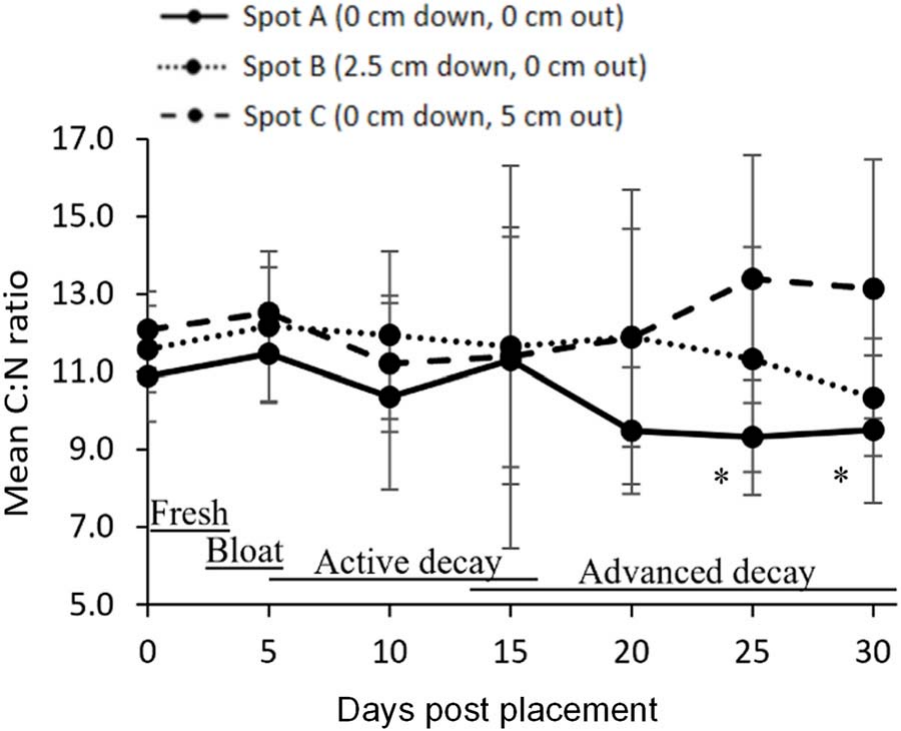
Mean (± standard error) values of carbon to nitrogen ratio (C:N) for three sampling spots adjacent to six human cadavers’ grave soils over the first 30 d of outdoor decomposition (PMI_30_) at the Forensic Anthropology Research Facility, San Marcos, Texas. Asterisk denotes a significant mean C:N change when compared to day 0, *P* < 0.05.

When examining the overall C:N ratio of gravesoils at the three body-adjacent sampling spots, the mean C:N over the course of decomposition decreased, given the relatively stable levels of carbon over the PMI_30_ period while the mean nitrogen content increased over the same time frame. This reduction in mean C:N from 10.9 to 9.3 was detected only for Spot A surface soils closest to the body on day 25 and day 25 of the study. During this time, the body was entirely in the advanced decay decomposition stage (Figure 3).

### *Gravesoils sampled over an extended PMI of up to 900 d (PMI*_*900*_)

**Figure 4:**
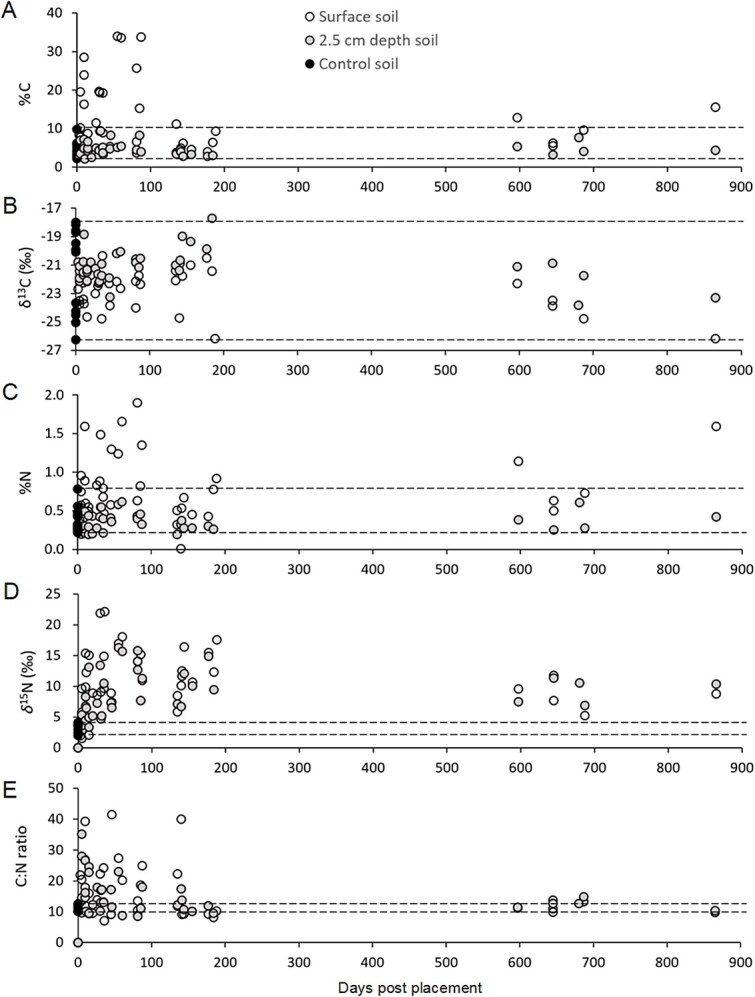
Measured values of soil percent carbon (A), δ^13^C (B), percent nitrogen (C), δ^15^N (D), and C:N (E) for individual cadaver gravesoils with various known postmortem intervals (PMI_900_) of outdoor decomposition at the Forensic Anthropology Research Facility, San Marcos, Texas (*n* = 43). (A) and (C): % ± standard error; (C) and (D): ‰ ± standard error. Dashed lines represent the range of control soil values compared with study samples.

Cadaver decomposition islands for the longer PMI_900_ study revealed differences between the two forms of carbon and nitrogen. Control soil %C had a narrow range of values (2.2%–9.8%) compared to the samples collected from gravesoils approaching 100 d of decomposition with values in excess of 30% C. These elevated surface gravesoils did not decline to the same range as control soils until 600–900 d of decomposition. Samples from those same CDI gravesoils taken at 2.5 cm depth showed no such spikes in %C with values between 2.1% and 9.3% throughout the extended PMI_900_ period, which aligned with the control soil range at the outset of the study (Figure 4A).

In contrast, control soil values for δ^13^C varied more significantly between −18.0‰ and − 26.3‰. This large variation in initial δ^13^C for the surface gravesoils mirrored the overall range of samples throughout the extended PMI period, although interestingly, the surface CDI gravesoils were depleted when compared to the corresponding CDI soils sampled at 2.5 cm depth (Figure 4B).

Values for both forms of nitrogen showed much narrower ranges of values when compared with their corresponding carbon values. Control soils had 0.2%–0.6% N, while surface CDI soils peaked at 1.9% at day 81, a four-to-five–fold increase, eventually declining back to control values between 600 d and 700 d postmortem. Surface gravesoils had consistently greater %N than the 2.5 cm depth soils at the same PMIs. Like the %C values, %N sampled at 2.5 cm depth did not fall outside the control soil value range so no significant elevation in %N was observed over the PMI_900_ study (Figure 4C).

Distinguishing itself from the trends found with δ^13^C, the control δ^15^N soils were more consistent with values from 2.0‰ to 4.1‰. This gives a range of ~2.0‰ for control soil δ^15^N, whereas the mean δ^13^C in control soil varied by ~8.3‰. With this tighter range of comparative control soil values, a clear trend of gravesoil enrichment for δ^15^N appeared almost immediately after cadavers were allowed to decompose, reaching more than 20‰ between 20 and 30 d of decomposition for surface gravesoils, and ~16‰ for the 2.5 cm depth soils around day 60. In general, the surface gravesoils were enriched when compared to the corresponding 2.5 cm depth soils over the PMI_900_ period. Both surface and 2.5 cm depth gravesoils did not lessen in δ^15^N to match that of the control soils and remained elevated throughout the PMI_900_ study (Figure 4D).

Examination of C:N in CDI gravesoils during 900-d PMI periods shows very narrow ranges for control soils (10.0–12.5) while surface soils C:N peaked from days 41 to 46 (>40), and 2.5 cm depth soils peaked at day 5 (>35). However, since these scatter plots show individual cadaver data points, this may be an outlier. If so, the next peak in 2.5 cm depth soils occurs at day 15 with a value of 24. Both surface and 2.5 cm depth gravesoils appear to return to control soil C:N values by 200 d (Figure 4E).

## Discussion

This study addressed three major questions: (1) Is there a quantifiable enrichment of δ^13^C and δ^15^N in CDI gravesoils over time, and if so, at what point in the early PMI is this enrichment first detectable, (2) do detectable levels of enriched δ^13^C and δ^15^N differ with increasing lateral and horizontal distances from the body, and (3) how long do these δ^13^C and δ^15^N enrichments persist over the longer PMI_900_? Current data have given clear evidence of how δ^13^C and δ^15^N are affected both temporally and spatially in human gravesoil CDIs.

Question 1 sought to determine whether δ^13^C and δ^15^N are enriched in human gravesoils as decomposition advances, and when it is detectable. Results indicate that of the two stable isotopes studied, δ^13^C and δ^15^N, significant enrichments were found with δ^15^N gravesoils in both PMI_30_ and PMI_900_ studies, providing evidence for its use in forensic taphonomy. While a cadaver contains both carbon and nitrogen, these substances are liberated into the surrounding CDI environment differently during decomposition. Benninger et al. [17] found gravesoils under decomposing porcines were significantly elevated in nitrogen first at day 14 of the experiment and remained elevated compared to control soils beyond 72 d. Carbon did not show a significant increase in gravesoils despite the large quantity of carbon contained in a body. This was ostensibly due to large fluctuations in carbon for both tissues and soils, with most carbon liberated from decomposition in the form of volatile gases, particularly CO_2_. The current study aligns with those findings whereby a significant amount of nitrogen, whether it be in total %N or δ^15^N, enters the CDI in appreciable quantities detectable in the PMI_30_ by days 10–15 during the active decay phase of decomposition. Active decay is when the greatest loss of mass from a cadaver occurs due to autolysis, bacterial degradation, and maggot larvae feeding so elevated levels of nitrogen in various forms were anticipated to increase during this stage. Soil δ^15^N is significantly elevated in the active decay stage of decomposition at days 10–15. Soil δ^13^C did not show any such enrichment over time.

The second component of the study addressed how far and how fast any stable isotope enrichment would be detectable not only at surface gravesoils directly adjacent to a cadaver, but also at a depth of 2.5 cm under the body or 5 cm out from the body. No clear pattern emerged with the three sampling spots for δ^13^C. Soil δ^15^N not only showed increased levels at Spot A collected at the surface and adjacent location, but also at Spot B (2.5 cm down) and Spot C (5 cm out from the body). However, the timing of these increases differed. Spot A showed significantly elevated δ^15^N by day 10, while Spots B and C experienced a delay in elevated δ^15^N until day 15. This stands to reason given that as tissues decompose and liquefy, they gradually purge from the body into the CDI and slowly permeate into the soil. Data reported in the current study are corroborated by studies using both porcines and humans reporting cadaver-derived nutrient data in CDI gravesoils [9–11, 13–17]. Cadaver and carcass nutrients permeate gravesoils horizontally and laterally over time, first detectable in surface soils with subsequent elevated nutrient levels detected at both lateral and horizontal distances from the body, dependent on soil type present.

Finally, extended period PMI_900_ gravesoils were studied to see how long these elevated levels of δ^13^C and δ^15^N persist, especially those CDIs where the body was removed. As with the early PMI_30_ study, δ^13^C in extended PMI_900_ period did not show any trends in enrichment because δ^13^C in the control soils had a wide range of values that prevented meaningful comparisons to the sampled gravesoils. Control samples for δ^15^N, however, had a very narrow range of values, making higher levels obvious in the data and more meaningful. Gravesoils sampled at both the CDI surface and 2.5 cm depth showed substantial increases very early in the PMI_900_ study that persisted in soils until 600–700 d of decomposition. Data reported in the current study supports other studies looking at longer PMI cadaver-derived nutrients in gravesoils. Data reported for forms of human tissue nitrogen in Tennessee show a return to control gravesoil levels by 700–800 d [11]. Stable isotopes analysed under decomposing beaver carcasses where elevated δ^15^N was detected 1 year after deposition at the site [12]. The discrepancy between elevated δ^15^N in beaver CDI gravesoil for 1 year and our study’s human gravesoil elevated δ^15^N detectable after 2 years is likely explained not only by the difference in soil types under the decomposing tissues but also the sheer size discrepancy between beavers and humans. With humans having on average two to four times the mass of an adult beaver, there is more soft tissue mass to decompose, so a greater volume of liquified nutrients is available to enter the adjacent soils and prolong nitrogen enrichment.

The current study data provide insights into the process through which nitrogen from decomposing organic material enters, is cycled through, and is liberated from the soil *via* the nitrogen cycle. In gravesoil, nitrogen enters the soil from liquefied decomposing tissues, shifting the soils from aerobic to anaerobic conditions [10]. Once in the soil, nitrogen can be converted through atmospheric nitrogen, ammonia, ammonium, nitrites, and nitrates by soil microbe metabolism. However, the soil’s oxygen content will determine how this cycle functions depending on whether the conditions are aerobic or anaerobic. In anaerobic conditions, ammonium cannot be converted to nitrites and nitrates by nitrifying microbes as this is an aerobic process; it is only when gravesoils revert to aerobic conditions that nitrification can resume [11]. This causes a lag in CDIs resuming the aerobic stages of the nitrogen cycle until further soil microbes gradually reintroduce more oxygen to the gravesoil. This delay in resuming aerobic metabolism explains the persistence of nitrogen, and in the current study specifically δ^15^N, by ~2 years. An extended period of high δ^15^N suggests such drastic levels of enrichment, ~20‰ for surface soil and 15‰ for 2.5 cm depth soil, indicating δ^15^N’s potential to establish the presence of a cadaver in a specific location for 2 years, even if the body has been moved to conceal it or if it has been scavenged and dispersed. This evidence has practical utility for forensic investigators even in the absence of human remains at the scene. However, it should be acknowledged that there is a conspicuous gap in the available CDIs sampled with no data available in the 200 to 600-d PMI range. Further studies of this nature should include additional data to provide a more comprehensive dataset. The current study data show that δ^15^N becomes rapidly enriched in gravesoils and persists to 600–700 d before returning to levels seen in control soils. Due to the wide range of both control and gravesoil δ^13^C values, it is difficult to draw any strong carbon isotope ratio conclusions from this aspect of the study.

In summary, δ^15^N has tangible promise as a forensic tool supplementing current trends in gravesoil science in determining not only the presence of decomposing remains, but also how long remains decomposed in that location. The temporal and spatial extension of the rates of δ^15^N liberation into CDI gravesoils is readily detected in the active decay phase of decomposition by days 10–15, depending on the spot sampled. This isotopic ratio enhancement endures for up to 2 years. Additional studies with greater sample sizes and with varied soil types could establish the utility of soil δ^15^N as another method in which forensic scientists and investigators can use with evidentiary value, a method that is easy to collect and relatively inexpensive compared to other chemical analyses currently used.
